# To be, or not to be, referred: A qualitative study of women from Burma's access to legal abortion care in Thailand

**DOI:** 10.1371/journal.pone.0179365

**Published:** 2017-06-12

**Authors:** Grady Arnott, Eh Tho, Niru Guroong, Angel M. Foster

**Affiliations:** 1 Faculty of Health Sciences, University of Ottawa, Ontario, Canada; 2 Cambridge Reproductive Health Consultants, Cambridge, Massachusetts, United States of America; 3 Mae Tao Clinic, Mae Sot, Thailand; 4 Independent consultant, Mae Sot, Thailand; PLoS, UNITED STATES

## Abstract

**Background:**

Reproductive health outcomes among women from Burma who live along the Thailand-Burma border demonstrate an unmet need for access to safe abortion services. In 2014, a multi-national team launched a collaborative three-year initiative to expand a program that refers eligible women for safe and legal abortion care to government Thai hospitals in Tak province, Thailand.

**Methods:**

Over a six-month period we conducted 14 in-depth open-ended interviews with women from Burma who were referred through the program or denied a wanted abortion after being deemed ineligible for referral by staff at the participating clinic. We analyzed the interviews for content and themes using both deductive and inductive techniques.

**Results:**

Women’s experiences accessing legal abortion care were positive and facilitated by appropriate options counseling, logistical support, and financial coverage. Five of the ineligible women we interviewed used traditional methods accessed on both sides of the border to self-induce an abortion and/or visited an untrained and unregulated provider.

**Discussion:**

Our findings highlight the need to redouble efforts to expand access to safe and legal abortion care for women from Burma residing in northern Thailand. Ensuring that women who are denied a safe and legal abortion receive harm reduction interventions and resources is critical.

## Introduction

Mass migration of people from Burma into neighboring countries is a result of a myriad of factors including decades of civil conflict, ongoing human rights violations, and the lack of socio-economic development and employment opportunities. These dynamics have led to the displacement of more than 1.5 million people into Thailand, a population that includes migrants with varied legal status and refugees in nine unofficial camps operating in the border region [1–2]. Maternal mortality and reproductive health indicators in northern Thailand reflect improved outcomes compared to Burma, and more specifically conflict-affected Eastern Burma where the maternal mortality ratio is estimated to be 1,000 deaths per 100,000 live births [3] However, among women from Burma living and/or seeking health services in Thailand, unmet contraceptive needs, increased risk of sexual violence and exploitation, and challenges with respect to accessing comprehensive reproductive health services persist and contribute to unintended pregnancy and unsafe abortion [3–9].

Abortion laws in Burma have remained unchanged since the establishment of the 1860 Burma Penal Code, which prohibits abortion in all cases except when terminating the pregnancy is required to save the life of the woman [10]. This exception is narrowly interpreted and procuring or providing an illegal abortion carries severe financial and criminal penalties for both the woman and the abortion provider. Unsafe abortion significantly contributes to maternal morbidity and mortality inside Burma [11–13] and it is well documented that women terminate pregnancies using unsafe practices including pummel massage and insertion of sharp objects on both sides of the border [7,11,14–17].

In Thailand abortion is regulated by Section 305 of the Thai Criminal Code and permitted in cases of life endangerment, rape, incest, and situations where the woman is 15 years of age or younger. The Thai Medical Council further defines eligibility on health grounds to include physical and mental health, including cases involving fetal anomalies as these conditions can impact a women’s mental health [18–19]. Yet, due to the cost of the procedure, restrictions on movement for migrants and refugees, cultural and linguistic differences between patients and providers, and both internalized and externalized stigma, women from Burma living in Thailand face significant challenges accessing safe abortion care, even in cases that align with the legal exceptions [7,20].

In April 2014, a multi-national, multi-disciplinary project team launched a three-year initiative to expand safe and legal abortion services and reduce mortality and morbidity from unsafe abortion along the Thailand-Burma border. The initiative follows the successful implementation of a pilot project that referred 24 eligible women from Burma living in Thailand for safe and legal abortion care at a Thai government hospital and covered the costs related to their procedure [20]. However, evaluation of the pilot also demonstrated that the vast majority of clients who were screened for a safe abortion referral were turned away because they did not meet the criteria for a legal abortion in Thailand. The formal evaluation did not include the perspectives of women with unintended or unwanted pregnancies who were either accepted or turned away for a legal referral.

This paper builds on previously published evaluation efforts and reports on the outcomes of women with unwanted or compromised pregnancies who were screened for safe and legal abortion care. As part of ongoing monitoring and evaluation of the extended initiative we conducted qualitative research to follow up with women who received, as well as those who were denied, a referral to a qualified Thai provider to learn more about the circumstances surrounding the pregnancy and pregnancy outcome.

## Methods

Through a qualitative study we aimed to document the experiences of patients who were both referred for and denied a referral for safe abortion care at a local Thai government hospital. We also aimed to identify strategies for improving the referral system along the Thailand-Burma border. Between October 2014 and April 2015 (inclusive) we conducted in-depth open-ended interviews with women from Burma residing in Thailand who presented at the Mae Tao Clinic (MTC). All clients who were screened by medics or counsellors and identified as being eligible for and wanting an abortion referral and/or having an unwanted pregnancy were able to participate in the study. We did not restrict eligibility based on age, marriage status, pregnancy history, or outcome of referral/non-referral.

As part of the established referral program, MTC used a stand-alone logbook to record patient information, including name, age, origin, gestational age, pregnancy history, maternal and/or fetal diagnosis (if applicable), and contact information, as well as whether or not the patient was a referral or non-referral case. Clinic staff recorded information in the logbook about all women who identified their pregnancies as unwanted during the preliminary counselling session. Clinic staff also recorded information about women with wanted pregnancies who were identified on intake and through subsequent medical examinations as having maternal and/or fetal health conditions and who ultimately wanted an abortion. Following counselling and determination of referral/non-referral status, clinic staff provided women with information about the purpose of the study and an invitation to participate. Staff informed referral clients that they would be contacted within one month of their scheduled procedure to discuss their experiences; women denied a referral would be contacted after three months’ time to discuss the outcome of the pregnancy. Clinic staff encouraged women who expressed interest in participating to contact the study team if their telephone number or other information changed. We followed-up with patients per the pre-determined timeline and scheduled interviews in a private location at a time convenient for the participant. To defray costs associated with transportation and missed labor we offered all participants 300 Thai Baht (approximately USD10 at time of the study) as well as snacks and drinks during the interview.

GA, a Canadian national with extensive international qualitative research experience, conducted all interviews in-person with the assistance of an interpreter fluent in Burmese or Karen. At the outset of the interview, we provided participants with a consent form and a lay language study information sheet to read or have read to them by the interpreter. All participants provided written or thumbprint consent and separate consent to audio-record the interview, per the approved protocol. GA took notes during the interview, debriefed with the interpreter after each interaction, and formally memoed following the interview to reflect on the researcher-participant-interpreter dynamics and initiate the analytic process. We transcribed verbatim and translated (to English) all interviews, which averaged an hour.

We developed, piloted, and revised two semi-structured interview guides for this study, one for referral and one for non-referral patients (see S1 File). We began all interviews by asking about the participant’s background and living situation before exploring her sexual, contraceptive, and pregnancy histories and the circumstances surrounding the index pregnancy. The guides then deviated; we asked referral clients about their experiences with options counselling, the referral process, and the care provided at the receiving hospital and we asked those deemed ineligible for a referral about their experiences with options counselling, reactions to the denial of the referral, subsequent experiences/actions related to the pregnancy, and pregnancy outcome/status. We finished all interviews by exploring ways in which the referral program could be improved, how women who are not eligible for referral could be better served, and how broader reproductive health service delivery could be enhanced.

Using transcripts, notes, and memos, we analyzed the interviews for content and themes and managed our data using NVivo (Version 10.2.0). GA served as the primary coder using both *a priori* (pre-determined) codes and categories based on the research questions and patient logbooks, as well as codes that emerged from the data [21–22]. Our approach was iterative such that data collection and analysis occurred simultaneously and regular team meetings guided the interpretation of the findings; we resolved differences through discussion.

Mae Tao Clinic approved the development and launch of the referral program and the overarching monitoring and evaluation plan. We received ethics approval from the Community Ethics Advisory Board at Mae Tao Clinic in October 2014 to conduct this study. In order to protect the confidentiality of our participants we have used pseudonyms throughout this article and have removed or masked all personally identifying information about participants and service providers. In the results section we present vignettes to showcase the overarching experiences of participants and illustrative quotes to showcase themes and subthemes.

## Results

### Participant sample and characteristics

**Fig 1:**
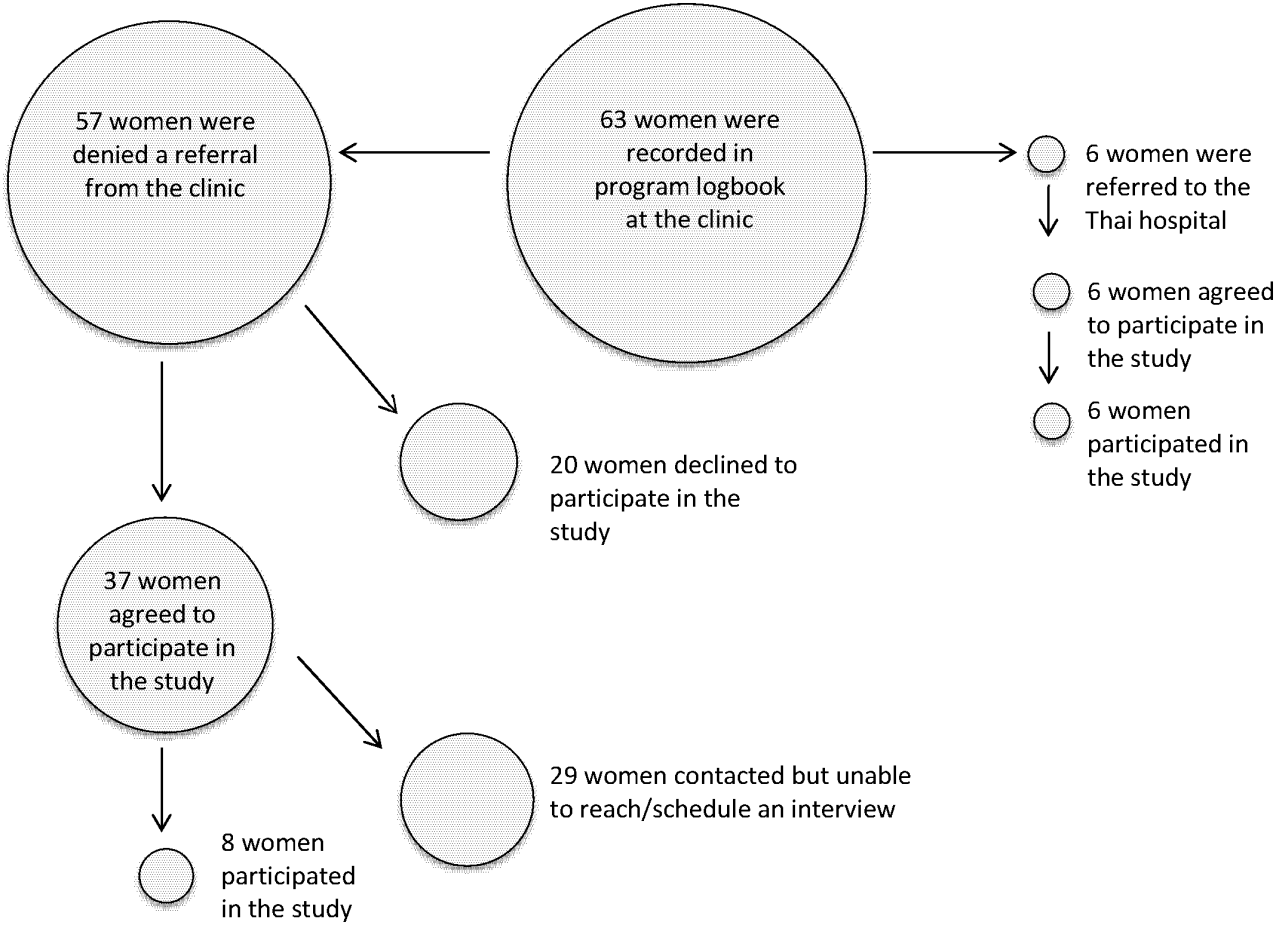
Participant sample.

Over the six-month data collection period we approached 42 women who were registered in the program logbook and expressed interest in participating in the study. Ultimately, we conducted in-person interviews with 14 clients; six women had received a referral for safe and legal abortion care and eight were denied a referral. We provide a schematic of our sample as Fig 1.

At the time of our interview, eight participants were living in the greater Mae Sot, Thailand area, five resided in Eastern Burma, and one was a refugee based in Mae La Camp. Participants ranged from 18 to 46 years of age, with an average age of 28. All but two of our participants were married, six had at least one child at the time of the interview, and two of these mothers were also grandmothers who were the primary caregivers for their grandchildren. Nine women first presented at the clinic at nine or less weeks’ gestation, three between nine and 12 weeks’ gestation, and one each at 22 weeks’ and 30 weeks’ gestation, respectively.

### Referrals hinged on physical maternal health conditions and fetal abnormalities

All six of the clients received referrals based on maternal health indications, including HIV and heart conditions, or detected fetal abnormalities or disease risk. Of the women we interviewed, three women described their pregnancies as wanted and/or planned. As demonstrated in Naw Thida’s story (Vignette 1), women with wanted pregnancies explained that their decision to terminate the pregnancy resulted from extensive discussions with husbands, family members, and support networks and was emotionally difficult. As one participant explained, ‘When I found out that the disease could transmit to my baby, I cried a lot and didn't want to have my baby anymore. The reason is I think about my baby; it doesn't mean that I don't want my baby.’

**Vignette 1. Naw Thida’s story**

*Following four years of seeking treatment at clinics and hospitals inside Burma, Naw Thida’s family grew worried of her deteriorating heart condition and raised funds to send her across the border to Thailand. Naw Thida presented at the border clinic in Mae Sot, Thailand with the hope of obtaining a referral to a Thai hospital for cardiac surgery. During the preliminary assessment she was informed that she was pregnant*.

*Naw Thida and her husband had been married for almost three years and eagerly anticipated starting a family. However, doctors and nurses explained that given the fragility and unpredictability of her condition, continuing the pregnancy would seriously threaten her life and would undermine the success of the surgery for which she had been approved. Naw Thida and her husband continued with counselling at the local clinic and reached out to other support organizations for several weeks. Although the decision was difficult, Naw Thida and her husband realized that her heart was not strong enough to sustain a pregnancy. With great sadness Naw Thida proceeded with the abortion before being transferred to Chiang Mai for heart surgery*. *(Interview conducted November 2014)*

The other three women described their pregnancies as unwanted. Although these women were able to obtain referrals on maternal or fetal health grounds, socioeconomic factors and relationship dynamics played a significant role in their decision to terminate the pregnancy. Women reported that they downplayed these factors in their counselling sessions, out of concern that they would not be referred for services.

### Referral clients reflected positively on their experiences with the program

Irrespective of the wantedness of the pregnancy, all referral clients expressed gratitude for being able to access a safe abortion performed by a trained provider in a hospital setting. As a 20 year-old primiparous woman who was referred for an abortion after detection of a lethal fetal abnormality explained:

My husband wanted to have baby, we were happy when I got pregnant. I enjoyed everything in my pregnancy, but when people told me I have to abort it we were upset. Now I am content because even if I did deliver, it will die; it would be hard for us to suffer through that. So the way medics deal with this for us [provide the abortion] was good for us.

In addition to the service itself, participants explained that the financial coverage provided by the program was critical. The program paid for all costs associated with consultation services, the procedure, medications, and post-abortion contraception (if desired) and averaged USD350 per patient, an amount that exceeds the average per capita annual income in the border region.

However, several participants explained that the project’s coverage of the costs associated with the abortion posed challenges with respect to disclosure of both underlying health conditions and the abortion itself. One participant living with HIV told family and friends that she went to the hospital following a miscarriage and that her husband paid for the treatment. As she explained, ‘If I told them that [the clinic/hospital] did it for me for free, they would know that I had the disease [HIV]…I protected myself by telling them that I had to pay.’

Although women were overwhelmingly positive about the program, several women reported that medical staff in the Thai hospital became angry or frustrated with them during their in-patient stay. As reported by one patient who received a later gestational age termination through induction with misoprostol:

The nurses lost tolerance because of the long day of treating me and they talked to me very hard. One of the nurses shouted at me because when she speaks to me in Thai I don’t understand what she says. Maybe she ran out of patience. My sister said that this nurse has a bad attitude, but I replied no they do not, it is because they have to take care of me every day so they lose patience. I can understand this very well.

Even in these circumstances patients reiterated their gratitude to the staffs at both the referral clinic and the receiving hospital and consistently expressed their desire for the program to expand and reach more women in need.

### Limited providers impedes access to timely abortion care

As illustrated in Htet Htet’s story (Vignette 2), most women were required to return to the hospital several times before obtaining their abortion procedure. During the study period, only one physician provided abortion care at the Thai hospital and those procedures were only offered one day a week. When this physician was out of town, on vacation, or called away for an emergency, abortion services were postponed. For undocumented women traveling across borders, this posed a significant challenge. One patient’s abortion was ultimately postponed by three weeks because of physician scheduling, a delay that pushed her into the second trimester of her pregnancy. Another referral patient’s care was postponed several weeks and she ultimately experienced a spontaneous abortion before she was able to come in for the procedure.

**Vignette 2. Htet Htet’s story**

*Htet Htet learned she was HIV positive after experiencing symptoms between her first and second marriage. Although she did not want more children her husband is opposed to her use of contraception and refuses to wear a condom. When she became pregnant for a third time she knew about dangerous options available in her community in Eastern Burma where unskilled providers will perform abortion for 60,000 kyat (approximately USD60). However, a member of her community support group for women living with HIV informed her about a program in northern Thailand that would refer her for a legal termination and cover the costs of the procedure. Htet Htet decided to cross the border to seek a safe abortion*.

*After arriving at the clinic Htet Htet was counselled by medics about ways to reduce mother-to-child transmission of HIV during pregnancy and childbirth. However, Htet Htet explained that in addition to her HIV status, her family’s economic situation did not put in her a position to parent for third time. After additional in-depth counselling she confirmed her decision to terminate the pregnancy and found comfort in being told that counselling staff would support her decision no matter the outcome. Htet Htet was referred to the Thai government hospital for blood testing and further counselling*.

*After being deemed eligible by clinicians at the Thai hospital Htet Htet was told to return the following Wednesday; the one providing clinician only offered abortion care one day a week. Without documentation to legally remain in Thailand, Htet Htet travelled back to her village and then returned one week later for the procedure as well as a post-abortion tubal ligation. She stayed in the hospital overnight and was discharged the following day without pain or complications*. *Reflecting on her overarching experience, Htet Htet explained:*

In reality people die from [unsafe abortion] and it is expensive to do too. I don't talk much about this with the community…but I want this [program] to develop more. I really thank this program. I am very happy to be treated equally in a country where I don't even belong.

(Interview conducted October 2014)

### Women denied referrals lack access to safe abortion care

We interviewed eight women with unwanted pregnancies deemed ineligible for legal abortion care by MTC counsellors and denied a referral to the Thai hospital. Consistent with women with unwanted pregnancies who did receive referrals, these participants reported that poverty, concerns about (un)employment, and the need to support their children and families shaped their decision to seek an abortion. Further, women denied referrals reported that relationship dynamics, young age, and feeling unprepared to parent also influenced their decisions.

Five of the women denied referrals reported during their interview that after being deemed ineligible they attempted to procure an abortion through an unregulated provider and/or through self-induction. Three of these women were successful in terminating the pregnancy, as is showcased in Naw Cell Khu’s experience (Vignette 3).

**Vignette 3. Naw Cell Khu’s story**

*Naw Cell Khu is in her early 20s and works 13-hour days, seven days each week at a local supermarket in Mae Sot, Thailand to support herself and her family. Her boss is strict; if she is late or misses work he deducts 500 baht (USD14) from her earnings. Thus she found herself in a challenging personal financial situation when she discovered she was pregnant. Although her partner at the time wanted her to continue the pregnancy, Naw Cell Khu felt she was too young to care for a child. This led to ongoing fighting and stress in their relationship*.

*The same day she learned about the pregnancy, Naw Cell Khu tried to induce an abortion using traditional Thai medicines for one week. When this strategy was unsuccessful, she ingested 15 sacks of Kay Thi Pan, a different herbal preparation widely believed to interfere with fertility, over a second week and then combined both traditional regimens with alcohol during a third week. She was confused and sad when the regimen did not work; many of her friends told her that they had used these traditional methods successfully in the past. Naw Cell Khu then went to MTC and asked if they could help her terminate the pregnancy. After medics explained that she was ineligible for a referral to the Thai hospital, they provided her with pamphlets about post-abortion care, which contained information about avoiding harmful practices and medications that can be used to induce abortion*.

*Naw Cell Khu knew she wanted to terminate the pregnancy and travelled across the border to seek services at an unmarked clinic in Burma. For 7,000 baht [approximately USD200] the clinic provided her with four misoprostol tablets. After a medic helped her administer two pills orally and insert two pills vaginally she was sent home with instructions to come back in a few days if the abortion was incomplete. Naw Cell Khu knew that she could not afford to buy more medicine from this clinic, so she returned to Thailand and hoped for the best*.

*Unfortunately, the misoprostol failed to induce an abortion and after several weeks she returned to MTC to see what more could be done. She explained to the medics that she still did not want to continue the pregnancy and detailed all the techniques she had used. She was told that the pregnancy was fine and she should stop trying to end it. As Naw Cell Khu explained, ‘I did all the methods, but it did not work. I felt hopeless*. *I thought I had done my very best already and I didn’t know where else to go.’*

*Eventually Naw Cell Khu paid a traditional birth attendant to give her pummel massage. The process took place over three days and left her bleeding an in extreme pain. Remembering the information contained in the pamphlet she returned to MTC for post-abortion care and was admitted overnight to the in-patient reproductive health service. Ultimately, Naw Cell Khu’s abortion experience took place over a three-month period and cost her 20,000 baht [approximately USD600], not including the pay check deductions she incurred for missing work*. *(Interview conducted April 2015)*

All of the women who reported attempting to end their pregnancy outside of the referral system reported that they had learned about providers and self-induction practices through their social networks and most availed themselves of services on both sides of the border. Women were generally aware that clandestine abortions pose significant health risks but as highlighted in Naw Cell Khu’s experience, safe services outside of the referral system remain limited. Even in cases where safer materials and medications are available through the black market and unmarked clinics, services are offered at exorbitant prices by unskilled providers constituting significant barriers and risks for women determined to end their pregnancies.

At the time of the interview, five of the women denied an abortion referral remained pregnant and all of these women were well into the second trimester of pregnancy or beyond. Two women reported that they had attempted to abort the pregnancy but were unsuccessful, as Wai Mar’s story illustrates (Vignette 4).

**Vignette 4. Wai Mar’s story**

*Wai Mar presented at MTC with concerns about a general loss of appetite and fatigue. Although she had recently discontinued her oral contraceptive pills due to side effects and safety concerns, she was surprised to learn she was three months pregnant. During her first pregnancy options counselling session she lied and said that the she already had three children and could not afford to look after a fourth; she hoped this strategy would allow her to get a referral for a legal abortion. However, the medics explained she was ineligible for a referral and told her that having children was a good thing and that after this pregnancy she could prevent future ones*.

*A domestic worker living in a small town in northern Thailand, Wai Mar was worried about raising a child in the current complex social and economic situation with her husband; he frequently drinks alcohol and becomes physically abusive. She was also concerned about the financial implications of parenting a child. A year earlier, Wai Mar self-induced an early abortion using traditional methods that she learned about from women in her community; she hoped she could terminate her current pregnancy in the same way. She repeatedly ingested packets of Kay Thi Pan and boiled ginger, but after four weeks it became clear that these techniques were not succeeding. When traditional methods failed her family and friends pressured her to keep the pregnancy so that she and her husband would have support as they age. She had heard of invasive unsafe methods that “women inside Burma” use to induce abortion but she was afraid to use them and became resigned to carrying the pregnancy to term*.

Right now what I am thinking about is my job. I worry that I will have to take a day off. I have to ride a bicycle to work and now I can't ride it anymore; it's getting heavier. Also I worry that I will not be able to keep my child in a good living standard like other children after giving birth…We have to pay for the house, electricity and water fees, which cost about 1,400 baht [approximately USD40] each month. Now when I give birth to my baby there won't be enough for us to spend on food and the house fees anymore. For me, I want to have a baby when the situation is good, but now, the situation is not good, so I don't want to have it yet. This is what is in my brain right now and I can’t think about the other things.

*Although Wai Mar reported that she would still have an abortion if she could have one safely in Thailand, she did not know of any options outside of the referral system. She planned to deliver at MTC later in the year*. *(Interview conducted March 2015)*

Consistent with the experiences of both Naw Cell Khu and Wai Mar, most of the women who attempted to induce an abortion returned to MTC on more than one occasion hoping for a referral. As indicated by another participant who tried to terminate the pregnancy on many occasions in between clinic visits, ‘I felt suffocated. I didn’t want the baby…[when I was told I was not eligible] I didn’t know what to do. I ran out of ideas.’ Unfortunately, irrespective of what techniques women had tried, none were subsequently referred and all were encouraged to embrace the situation and plan for the pregnancy. Other than information about post-abortion care, which was only provided to one woman, limited resources and options to support clients with unintended and/or unwanted pregnancies exist in this setting.

Although poverty and financial constraints played a major role in women’s motivations for seeking an abortion, most of the women denied a referral explained that if they knew where they could get a safe(r) abortion without criminal penalty they would find the funds. As exemplified by one participant who was pregnant at the time of the interview, ‘I would have gone if I knew the place. If it was a clinic I would have gone, but not to other places. I think it would be safe at the clinic. I would still do it [terminate the pregnancy] if I could.’ During the interviews, several participants inquired as to whether or not it was still possible for them to have an abortion and wanted information about where they could go to seek services outside the referral system.

## Discussion

Improving women’s experiences and access to safe abortion care in legally restricted settings has been addressed using public health, human rights, and social justice interventions [20, 23–24]. However, unsafe abortion still accounts for 13% of all maternal deaths globally [25] and 60–65% of all abortions that take place in South East Asia are defined as unsafe [26]. Our findings are congruent with a body of research that consistently demonstrates that, irrespective of legal status, eligibility criteria, or availability of safe options, women will continue to seek to terminate unwanted pregnancies [11,14–17,25,27]. Liberalization of the abortion laws in both Burma and Thailand would serve as an important step in addressing women’s comprehensive reproductive health needs.

However, even in the absence of legal reform, the safe abortion referral program along the Thailand-Burma border has reduced significant challenges that eligible women would otherwise face when attempting to access services at Thai government hospital facilities, including socio-linguistic, financial, documentation, and transportation barriers. That patients are grateful for the program and being able to obtain safe care is not surprising given the grim alternatives. Even when faced with hostile treatment or considerable delays, women remained satisfied with their care. However, the experiences of women who were referred suggest that continued in-service training for health care professionals at both the referring and the receiving facility is warranted. Further, the limited schedule during which abortion care is offered in the receiving hospital is especially challenging for migrant and undocumented women. Moving forward, alternatives that allow referrals to take place on multiple days or establishing networks with other abortion providers in the northern Thailand region for more flexibility could greatly improve the referral and abortion experiences of eligible women.

However, the most significant challenge with the program relates to the number of women deemed ineligible for a legal referral. Indeed, during the six-month study period less than 10% of the patients documented in the program logbook with unintended and/or unwanted pregnancies were referred for legal care. Exploring ways to work within the legal constraints to refer more women for safe abortion care is a priority. Importantly, no women presenting at MTC were referred to the Thai hospital for mental health indications, although abortions are commonly performed for this reason in Thai hospitals in other regions of the country [18]. Additional efforts to work with MTC medics and counsellors to utilize the full range of eligibility criteria could have a significant impact on access.

Yet even with expanded access through the referral system, it is likely that a significant proportion of women will ultimately be deemed ineligible for legal care. The experiences of our participants indicate that few options and resources are made available to them, even though it is well-established that many women in this region often turn to unsafe alternatives [5–7,11,16]. The experiences of our participants who were denied an abortion referral are consistent with this larger trend and reveal that women may combine safer but less effective methods with dangerous but more effective techniques. Routinely and consistently providing women with information about post-abortion care—and reiterating to women that seeking such care is legal—could help women who engage in dangerous practices better manage the health consequences.

However, identifying avenues to increase access to safe(r) abortion care in this region is also pressing. In recent years, telemedicine services such as Women on Web and Women Help Women have provided medically-eligible women living in settings where abortion is legally restricted with the gold standard medication abortion regime of mifepristone/misoprostol. Although access to the internet, literacy (or a literate interlocutor), and a physical address to receive a shipment is required, increasing awareness of this service could help women with early unwanted pregnancies obtain a safe abortion.

Further, misoprostol—a prostaglandin E_1_ analogue used for a variety of obstetric and gynecological indications—is widely available along the border as showcased in Naw Cell Khu’s story. Misoprostol can be used as a single abortifacient in the first nine weeks of pregnancy at 70%-90% efficacy [28]. Although not as effective as the mifepristone/misoprostol regimen, use of misoprostol alone is much safer and more effective than the abortion methods currently used by women outside of safe and legal clinical settings in this region. As mifepristone is not currently available in northern Thailand, identifying avenues for expanding access to information about evidence-based misoprostol protocols to both health care workers and individual women may prove to be a successful strategy for reducing harm from unsafe abortion.

## Limitations

Using qualitative methods allowed for an exploration of the experiences of clients who used or were turned away from the safe abortion referral system and their opinions on how to improve the program. Due to the highly transient nature of the study population combined with the stigma and illegality surrounding abortion in this context, we were only able to interview 14 women. Although we identified consistent themes, future research would benefit engaging with a larger number of women over a longer period of time. Further, as the program expands it will be important to include women who interact with all organizations involved with the program. As with all qualitative research, our results are not intended to be representative or generalizable. However, we believe that the findings are transferable beyond the bounds of the immediate study population and our rigorous design enhances both the credibility and trustworthiness of the findings.

## Conclusion

The findings from this qualitative study demonstrate that women’s reproductive health experiences are greatly impacted by access to comprehensive reproductive health services, including safe and legal abortion care. The safe abortion referral program along the Thailand-Burma border has established an important mechanism for women deemed eligible to access legal abortion care in Thailand and merits expansion and scale-up. Meeting the needs of women with unwanted pregnancies deemed ineligible for legal abortion care remains a significant gap in service delivery in the region. Therefore, identifying ways to reduce harm from unsafe abortion and share information regarding a women’s right to post-abortion care appears warranted.

## Supporting information

S1 File Interview guide. (PDF)
