# Supplementary material for: To be, or not to be, referred: A qualitative study of women from Burma's access to legal abortion care in Thailand
Source: PLoS One. 2017 Jun 12;12(6):e0179365. doi: 10.1371/journal.pone.0179365 (PMC5467911; doi:10.1371/journal.pone.0179365)
Supplement: S1 File — (PDF) [file pone.0179365.s001.pdf]

Safe Abortion Referral Project
In-depth interview guide for women who received a referral

Introduction
Thank you again for participating in the study. Let me begin by introducing myself [introduction]. I'd also like [name of interpreter] to introduce herself [introduction]. You can speak in English, Karen, or Burmese. Please feel free to speak openly and use any language or words that you are comfortable. There are no right or wrong answers. Again, you can choose to stop participating in this discussion at any time and you can also choose to not respond to any question you don't want to answer. With your permission, I will be turning on the recorder now.

General information
1. I'd like to begin by asking you tell me a little bit about yourself
2. Please tell me about your current living situation
3. What are some routine activities that you do?

General sexual, contraceptive, reproductive and pregnancy history
1. Please tell me about your reproductive health overall
2. Please tell me about the most important reproductive health events that you experienced
3. Please tell where you go to get reproductive health care
4. Please tell me [more] about your experience(s) with MTC

Experience with the safe abortion referral
1. To begin, please tell me about the circumstances surrounding the pregnancy
2. Now I'd like you to tell me about your consultation session and experience with options counselling offered through MTC
3. Can you tell me about your experiences throughout the safe referral process?
4. How did you feel about the services you received at [hospital]?
5. Do you have any other opinions about, or experiences with, using the safe abortion referral system that you would like to share with me?

Opportunities for change
Thank you so much – we are almost done! I'd like to ask you now about how improvements can be made to the safe abortion referral program along the Thailand-Burma border
1. What do you think about your experience using the referral system in general?
2. Would you recommend MTC for pregnancy options counselling to other women?
3. Would you recommend [hospital] for safe abortion to other women who are eligible?
4. What do think could be done to improve the safe abortion referral program for women along the border?
5. What do think could be done to improve reproductive health service delivery along the border more generally?

Wrap-up
Thank you very much for taking the time for speaking with me today. That's all the questions I have.
1. Is there anything that you would like to add?
2. Is there anything that I should have asked but didn't?
3. Do you have any questions for me?

Safe Abortion Referral Project
In-depth interview guide for women denied a referral

Introduction

Thank you again for participating in the study. Let me begin by introducing myself [introduction]. I'd also like [name of interpreter] to introduce herself [introduction]. You can speak in English, Karen, or Burmese. Please feel free to speak openly and use any language or words that you are comfortable. There are no right or wrong answers. Again, you can choose to stop participating in this discussion at any time and you can also choose to not respond to any question you don't want to answer. With your permission, I will be turning on the recorder now.

General information

1. I'd like to begin by asking you tell me a little bit about yourself
2. Please tell me about your current living situation
3. What are some routine activities that you do?

General sexual, contraceptive, reproductive and pregnancy history

1. Please tell me about your reproductive health overall
2. Please tell me about the most important reproductive health events that you experienced
3. Please tell where you go to get reproductive health care
4. Please tell me [more] about your experience(s) with MTC

Experience with the safe abortion referral

1. To begin, please tell me about the circumstances surrounding the pregnancy
2. Now I'd like you to tell me about your consultation session and experience with options counselling offered through MTC
3. Can you tell me about the outcome of your pregnancy after not being able to receive a funded referral?
4. Do you have any other opinions about, or experiences with, your pregnancy that you would like to share with me?

Opportunities for change

Thank you so much – we are almost done! I'd like to ask you now about how improvements can be made to the safe abortion referral program along the Thailand-Burma border

1. Would you recommend MTC for pregnancy options counselling to other women?
2. What do think could be done to improve services for women who have unintended or unwanted pregnancies, but are not able to use the safe abortion referral program?
3. What do think could be done to improve reproductive health service delivery along the border more generally?

Wrap-up

Thank you very much for taking the time for speaking with me today. That's all the questions I have.

1. Is there anything that you would like to add?
2. Is there anything that I should have asked but didn't?
3. Do you have any questions for me?
